# *Campylobacter jejuni* Biofilm Formation Under Aerobic Conditions and Inhibition by ZnO Nanoparticles

**DOI:** 10.3389/fmicb.2020.00207

**Published:** 2020-03-10

**Authors:** Xian Zhong, Qingping Wu, Jumei Zhang, Zonghao Ma, Juan Wang, Xiang Nie, Yu Ding, Liang Xue, Moutong Chen, Shi Wu, Xianhu Wei, Youxiong Zhang

**Affiliations:** ^1^State Key Laboratory of Applied Microbiology Southern China, Guangdong Provincial Key Laboratory of Microbial Culture Collection and Application, Guangdong Open Laboratory of Applied Microbiology, Guangdong Institute of Microbiology, Guangdong Academy of Sciences, Guangzhou, China; ^2^Hong Zheng Dao (China) Traditional Chinese Medicine Research Company Ltd., Guangzhou, China; ^3^College of Food Science, South China Agricultural University, Guangzhou, China

**Keywords:** *Campylobacter jejuni*, biofilm, mixed culture, pure culture, ZnO nanoparticles

## Abstract

*Campylobacter jejuni* is a major foodborne pathogen worldwide. As it forms biofilms, it can become a persistent contaminant in the food and pharmaceutical industries. In this study, it was demonstrated that *C. jejuni* could make more biofilm in aerobic conditions than in microaerobic conditions, and only 13.9% *C. jejuni* entered coccus (a VBNC state) under microaerobic conditions; however, the rate increased to 95.5% under aerobic conditions. *C. jejuni* could form more biofilm in mixed culture with *Escherichia coli* or *Pseudomonas aeruginosa* than in pure culture. Scanning electron microscope results showed that *C. jejuni* retained its normal spiral shape under aerobic conditions for 48 h by forming crosslinks with the aerobic and facultative anaerobic bacteria. Additionally, culture medium containing 0.5 mg/ml ZnO nanoparticles inhibited biofilm formation. Our results provide information on a new approach to controlling contamination via *C. jejuni.*

## Introduction

*Campylobacter jejuni* is a zoonotic pathogen, and it is a major cause of foodborne diseases all over the world (Ica et al., 2012). Planktonic *C. jejuni* is extremely susceptible to multiple stressors (Lee et al., 2019; Oh et al., 2019), especially to oxygen pressure in the environment, as aerotolerance factors heavily in the transmission of *C. jejuni* to humans via foods under aerobic conditions (Euna et al., 2015; Oh et al., 2018). *C. jejuni* can be observed in a viable but non-culturable (VBNC) state *in vitro* (Magajna and Schraft, 2015; Stetsenko et al., 2019). However, it is easy to acquire *C. jejuni* from water, soil, and so on (Teh et al., 2014). It is also puzzling that although planktonic *C. jejuni* has a weak ability to survive multiple stressors in the environment, it can cause foodborne diarrheal illness (Karki et al., 2019; Kim et al., 2019). Research on *C. jejuni* infections is rare compared to research on other foodborne pathogens, such as *Salmonella* and *Listeria monocytogenes*. However, previous research has shown that *C. jejuni* biofilms can resist harsh environments, which enhances its transmission ability (Teh et al., 2010). Natural bacteria biofilms involve complex cellular communities and they have major implications regarding pathogenesis (Jothiprakasam et al., 2017).

Many bacteria have the ability to form biofilms, which involves encasing the bacteria in a sticky polymer (Kwiecinski et al., 2019). Bacterial biofilms confer survival advantages to the bacteria because they protect the bacteria from environmental stressors, such as ultraviolet light, dehydration, and treatment with antimicrobial and sanitizing agents (Ica et al., 2012). This makes their elimination challenging (Bae and Jeon, 2013). The formation of *C. jejuni* biofilm can be induced by many internal and external factors, such as the surface properties of the material that the bacteria is on, the temperature, and oxygen levels (Wu et al., 2016). In nature, biofilm formation involves a mixture of bacteria (Otto, 2014; Solano et al., 2014), which can help to provide *C. jejuni* protection during the transmission process. Research on mixed-culture bacterial biofilms is very popular; some research has shown that the presence of mixed populations of bacteria could enhance the number of *C. jejuni* (Sanders et al., 2007). Most studies mainly focus on genes related to biofilm formation, surface adhesion, colonization, movement, and regulation of extracellular secretions–however, it is difficult to quantify the effects of multiple genes and sequence variations (Pascoe et al., 2016). Research has shown that the formation of *C. jejuni* monoculture biofilms is difficult (Joshua et al., 2006; Hanning et al., 2008). The current study simulated the conditions (Oh et al., 2018) involved in the *C. jejuni* infection of humans (at 37°C under aerobic conditions representing the natural atmosphere) and explored the mechanisms related to infection.

Once a biofilm is formed, it is hard to remove and can cause persistent contamination. Mechanical removal is used to control biofilm formation in the food industry, but this procedure cannot be used to solve the dead angle problem. Additionally, use of surfactants and strong detergents, as well as acids, alkalis, and oxidizing agents, can lead to more serious biofilm adhesion, and it is difficult to ensure that the chemicals penetrate the biofilm to kill the bacteria (Wu et al., 2016). Zinc oxide (ZnO) is “generally recognized as safe” (GRAS) according to the U.S. Food and Drug Administration (21CFR182.8991). The high surface-to-volume ratio of ZnO nanoparticles allows for better interaction with, and toxicity toward, bacteria. This study is the first study to explore the use of ZnO nanoparticles to control *C. jejuni* biofilm formation.

## Materials and Methods

### Preparation of Strains

We used the standard *C. jejuni* strain CICC 22936, two *C. jejuni* isolates (3375A and 351-2B, which have a strong and weak ability to form biofilms, respectively) that were isolated by our laboratory from retail food samples in China. The *C. jejuni* strains were identified by conventional biochemistry and duplex PCR methods (Rui et al., 2007), as has been reported by our laboratory (Zheng et al., 2014; Zhong et al., 2016). *Pseudomonas aeruginosa* ATCC15442 and *Escherichia coli* ATCC12900 were used to co-culture with *C. jejuni.*

### Biofilm Measurement

Two strains (one with a strong ability to form biofilms and one with a weak ability) were selected using crystal violet staining. The difference in biofilm formation between *C. jejuni* in pure culture and mixed culture with *E. coli* (facultative anaerobic bacteria) or *P. aeruginosa* (aerobic bacteria) was assessed. Additionally, three concentrations of ZnO nanoparticles were added to the medium to study the inhibition effect of ZnO nanoparticles on biofilm formation (described below).

The concentration of all the bacterial suspensions was adjusted to 0.5 McFarland using Mueller-Hinton broth (Huankai, Guangzhou, China) by assessing the optical density at 590 nm. For some of the experiments, 0.5, 5, or 50 mg ZnO nanoparticles (30 ± 10 nm; Mclean Biochemical, China) were added to liquid dispersion bottles containing 10 ml MH broth. The suspensions were then sterilized at 121°C for 15 min and used in subsequent experiments.

#### Biofilm Measurement by Crystal Violet Staining

Each bacterial suspension (*C. jejuni* CICC 22936 and *E. coli* cultured individually or together; *C. jejuni* CICC 22936 and *P. aeruginosa* cultured individually or together; and mixed culture of *C. jejuni* CICC 22936 and *P. aeruginosa* or *E. coli* treated with three concentrations of ZnO nanoparticles, 0.005, 0.05, and 0.5 mg/ml, respectively) was added to 96-well plates (Corning, United States). Each well contained 20 μl bacterial culture and 180 μl MH broth. The bacteria were cultured at 37°C for 48 h under aerobic conditions. Each sample underwent eight identical parallel tests. The 96-well plates were gently rinsed three times with sterilized water and dried at 55°C for about 30 min. Staining was conducted by adding 200 μl 0.1% crystal violet dye (Jinsui Biological, China) to each well for 10–15 min. The wells were washed with sterilized water and dried at 55°C. Next, 300 μl eluent (80% ethanol and 20% acetone) was added and the biofilm was detected by assessing the optical density at 590 nm (Reeser et al., 2007). The above processes were also conducted in test tubes for easier visualization of the biofilms.

#### Biofilm Measurement Using Scanning Electron Microscopy (SEM)

First, 8-mm cell climbing film (Wohong Biological, China) was added to 48-well plates (Corning, United States). Next, 100 μl *C. jejuni* (pure culture) or 50 μl *C. jejuni* with 50 μl *E. coli* or *P. aeruginosa* bacterial culture (mixed cultures) was added to each well along with 900 μl MH broth to ensure that each sample had an equal number of cells. The bacteria were cultured at 37°C for 48 h in aerobic or microaerobic (5% O2, 10% CO2, and 85% N2) conditions. The excess liquid was removed, the plates were rinsed twice with phosphate-buffered saline, and the bacteria were then immediately fixed with 2.5% glutaraldehyde and dehydrated using a graded series of ethanol concentrations prior to observation using SEM (S-3000N; Hitachi, United States) (Joshua et al., 2006).

#### Biofilm Measurement Using Confocal Laser Scanning Microscopy

First, 200 μl ZnO nanoparticles suspension was added to 24-well plates and 14-mm-diameter cell climbing film was used as the carrier. Next, 200 μl *C. jejuni* (pure culture) or 100 μl *C. jejuni* with 100 μl *E. coli* or *P. aeruginosa* bacterial culture (mixed cultures) were added to each well along with 1800 μl MH broth. The bacteria were cultured at 37°C for 48 h in aerobic conditions. Excess liquid was removed by pipetting, and the 24-well plates were washed with sterile water three times. Thereafter, 500 μl of 0.1% SYTO 9 Green Fluorescent Nucleic Acid Stain (Thermo Scientific, United States) was added and incubated at 37°C for 30 min and 500 μl of 10% propidium iodide (PI) was added and incubated at 4°C for 15 min in the dark (Ica et al., 2012). Residual dye was removed and the plates were washed prior to observation using confocal laser scanning microscopy (LSM 700; Carl Zeiss, Germany).

## Results

### Biofilm Formation of *C. jejuni* in Pure Culture

**FIGURE 1:**
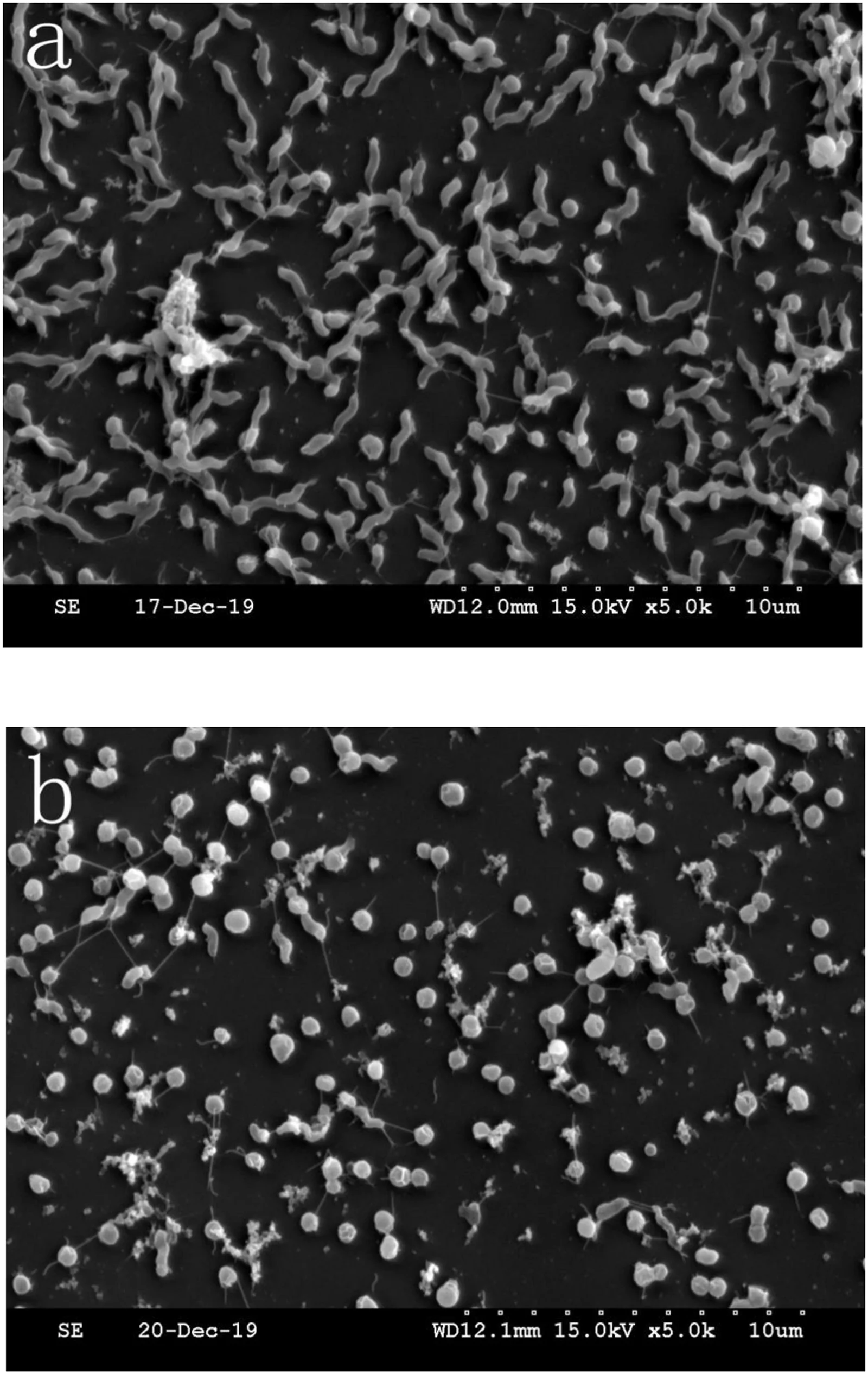
Representative scanning electron microscopy (SEM) images. **(a)** SEM image of *C. jejuni* cultured under microaerobic conditions. **(b)** SEM image of *C. jejuni* cultured under aerobic conditions.

*Campylobacter jejuni* biofilm formation was compared between aerobic and microaerobic conditions in pure culture. There were two morphology types of cells, the number of which was quantified in a fixed area of the microscope. As shown in Figures 1a,b, repeated the count three times in each different culture condition 86.1% *C. jejuni* had a normal spiral shape and 13.9% *C. jejuni* entered coccus (a VBNC state) under microaerobic conditions. Only 4.5% *C. jejuni* had a normal spiral shape, and 95.5% *C. jejuni* entered coccus under aerobic condition.

The crosslinking substances were increased in Figure 1b compared to Figure 1a, indicating that there was more *C. jejuni* biofilm formation under aerobic conditions than under microaerobic conditions.

### Biofilm Formation of *C. jejuni* in Mixed Culture With *E. coli* or *P. aeruginosa*

**FIGURE 2:**
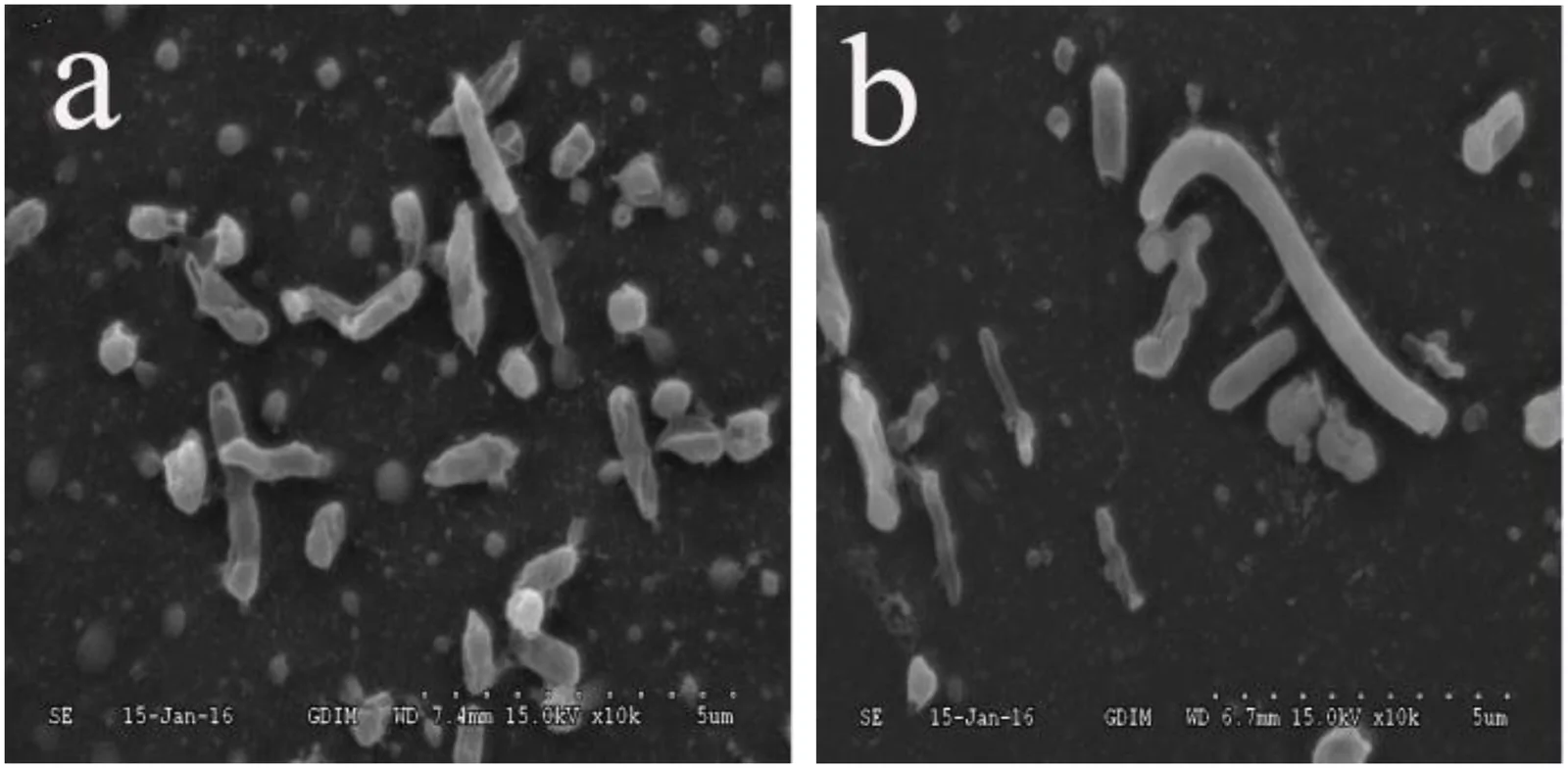
**(a)** SEM image of *C. jejuni* co-cultured with *P. aeruginosa* under aerobic conditions at 37°C for 48 h. **(b)** SEM image of *C. jejuni* co-cultured with *E. coli* under aerobic conditions at 37°C for 48 h.

**FIGURE 3:**
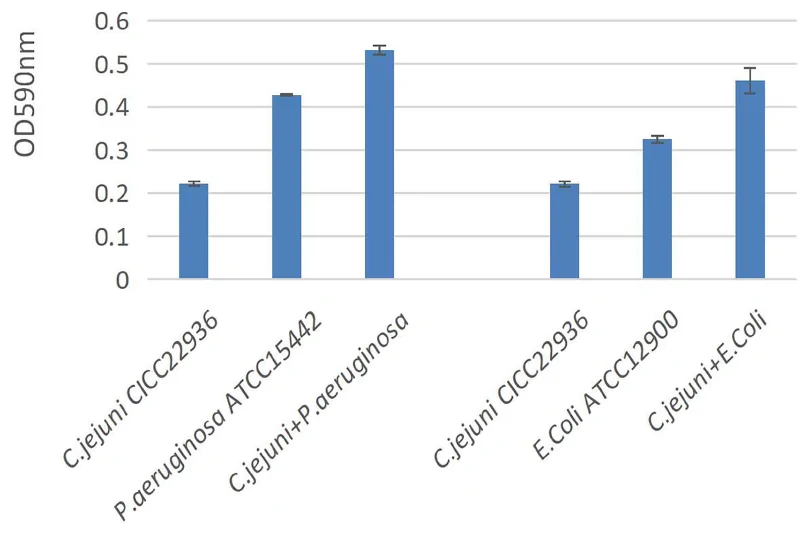
The *C. jejuni* biofilm formation under aerobic conditions was compared between pure and mixed cultures. Biofilm formation in 96-well plates under aerobic conditions at 37°C for 48 h was assessed by crystal violet staining and optical density (OD) assessment. Experiments were performed in triplicate on three separate occasions. The assay was carried out in triplicate, and one representative experiment of three experiments is shown with error bars.

The *C. jejuni* biofilm formation under aerobic conditions was compared between pure and mixed cultures. The *C. jejuni* in pure culture had a changed morphology, as shown in Figure 1b. However, in mixed culture with *E. coli* or *P. aeruginosa*, it retained its normal spiral shape, as shown in Figures 2a,b. In mixed culture, *C. jejuni*, which had a normal spiral shape, was near *E. coli* or *P. aeruginosa*. Additionally, the crystal violet staining (in both the test tubes and 96-well plates) demonstrated that the biofilm formation of *C. jejuni* increased in mixed culture with *E. coli* and *P. aeruginosa* compared to in pure culture, as shown in Figure 3.

Crystal violet staining was conducted to detect biofilms of *C. jejuni*, *P. aeruginosa*, and *E. coli* pure or mixed cultures in 96-well plates in aerobic conditions at 37°C for 48 h. The co-cultured *C. jejuni* formed significantly more biofilms than the *C. jejuni* in pure culture.

**FIGURE 4:**
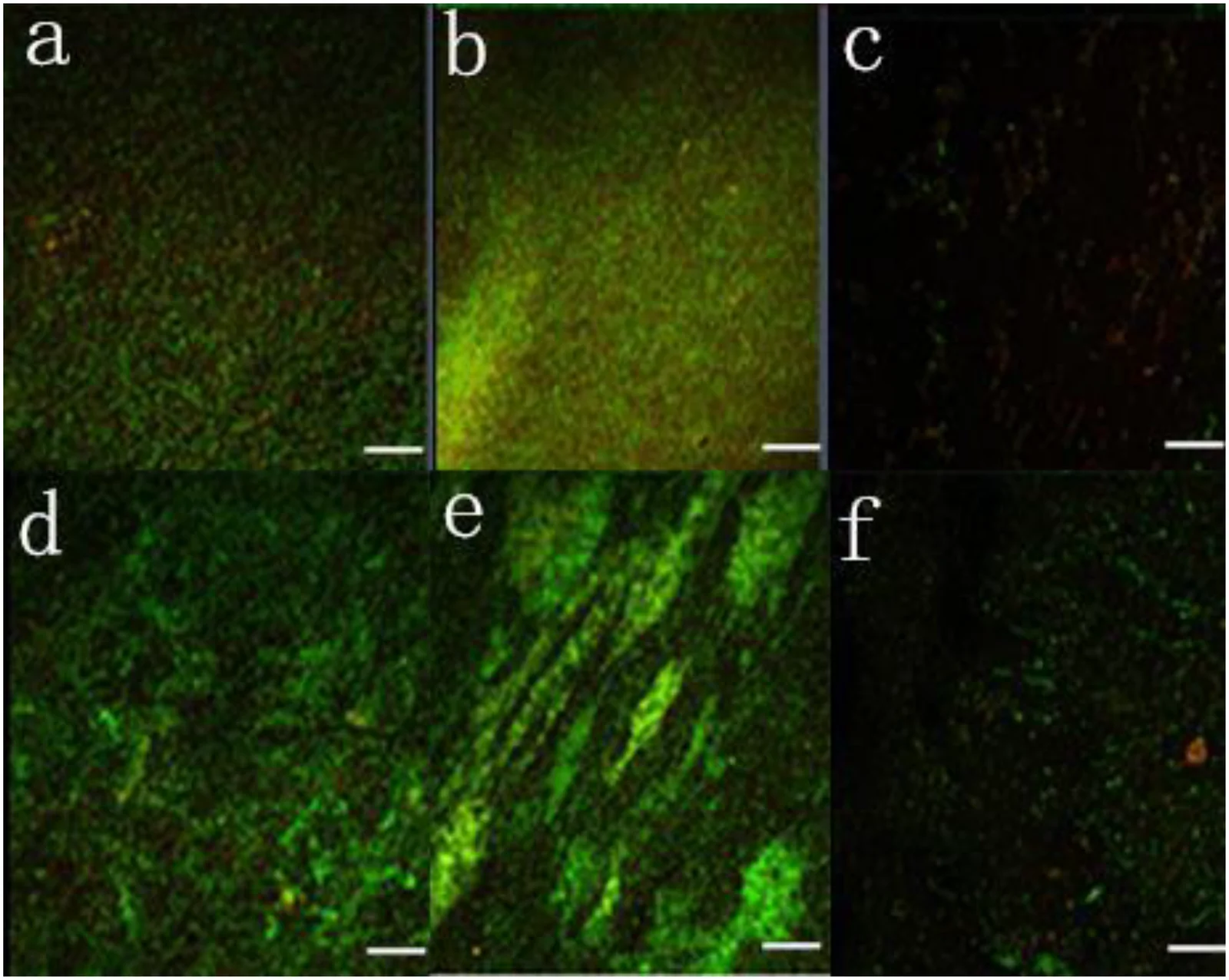
Confocal laser scanning microscopy images of live and dead bacteria in biofilms under aerobic conditions. **(a)** *P. aeruginosa*, **(b)** *C. jejuni* mixed with *P. aeruginosa*, **(c)** *C. jejuni* mixed with *P. aeruginosa* and treated with 0.5 mg/ml ZnO nanoparticles, **(d)** *E. coli*, **(e)** *C. jejuni* mixed with *E. coli*, and **(f)** *C. jejuni* mixed with *E. coli* and treated with 0.5 mg/ml ZnO nanoparticles.

In addition, biofilms in pure and mixed cultures were compared using confocal laser scanning microscopy with SYTO9 and PI staining, as shown in Figure 4. The fluorescence signal was stronger in the mixed cultures than in the pure culture, indicating that the number of live cells in the mixed culture biofilms was greater than in the pure culture biofilms.

### Effect of ZnO Nanoparticles on Biofilm Formation

**FIGURE 5:**
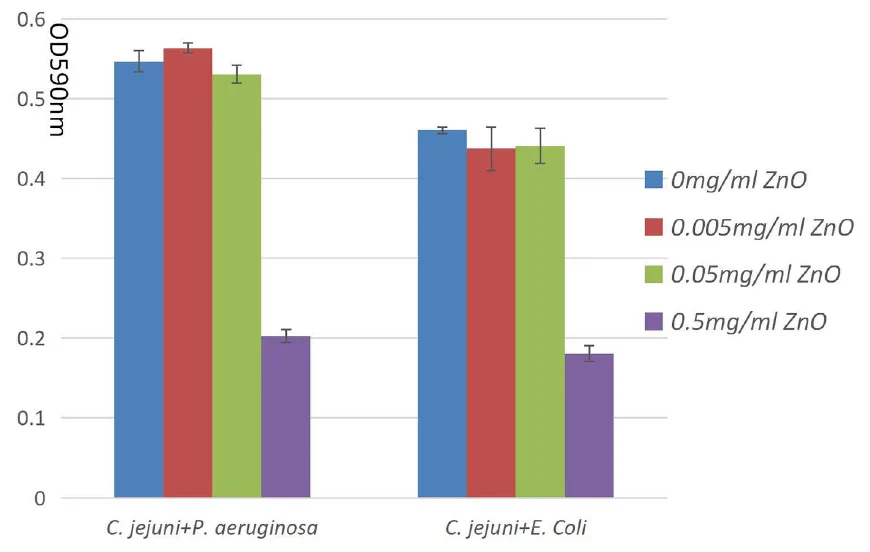
Biofilm formation of *C. jejuni* mixed culture with *E. coli* or *P. aeruginosa* treated with 0, 0.005, 0.05, 0.5 mg/ml ZnO nanoparticles in 96-well plates was assessed by crystal violet staining and optical density (OD) assessment. The assay was carried out in triplicate.

**FIGURE 6:**
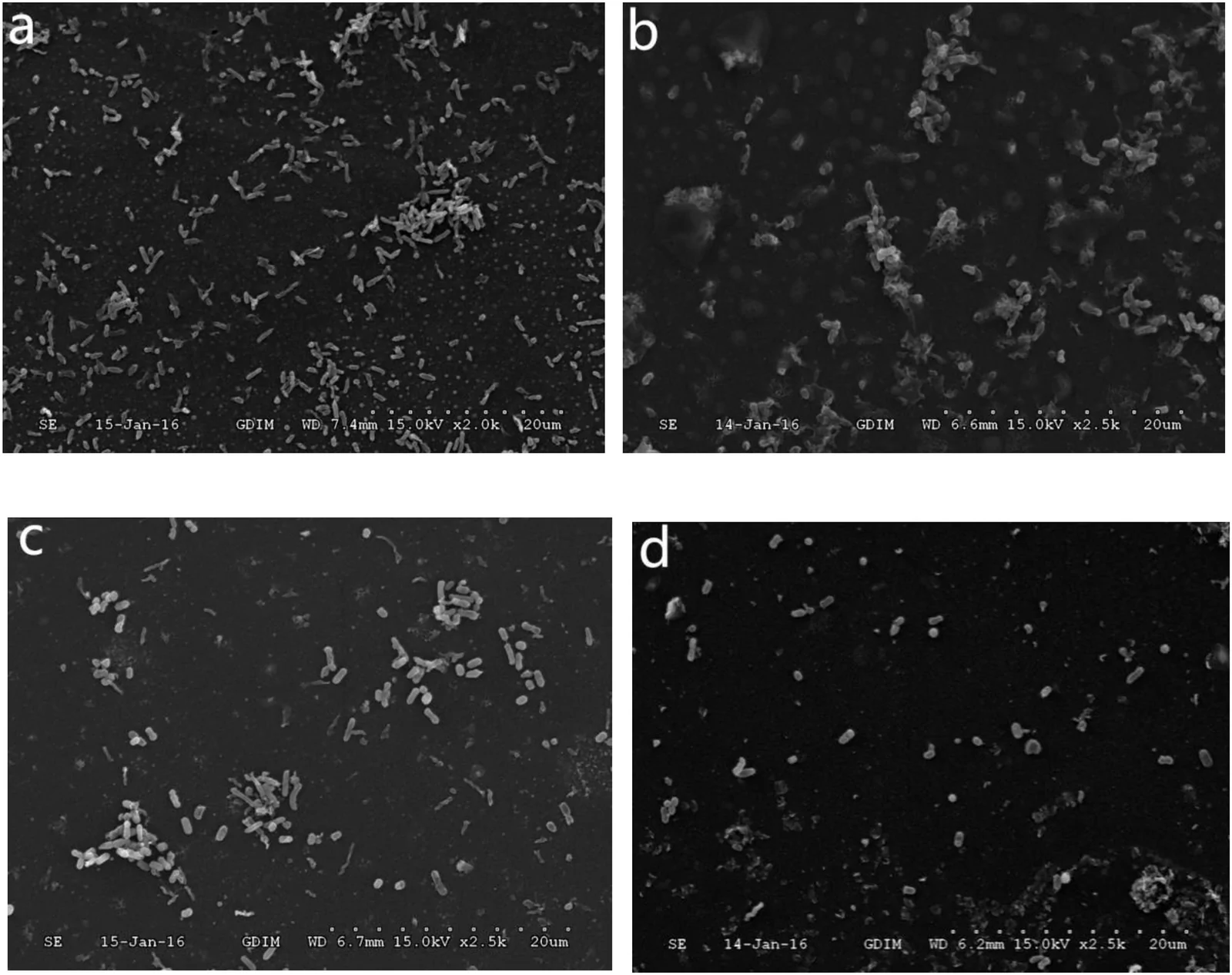
Scanning electron microscopy (SEM) images of *C. jejuni.* **(a)** Mixed culture of *C. jejuni* and *P. aeruginosa*, **(b)** Mixed culture of *C. jejuni* and *P. aeruginosa* treated with 0.5 mg/ml ZnO nanoparticles, **(c)** Mixed culture of *C. jejuni* and *E. coli*, **(d)** Mixed culture of *C. jejuni* and *E. coli* treated with 0.5 mg/ml ZnO nanoparticles.

The ZnO nanoparticles inhibited the biofilm formation of *C. jejuni* under aerobic conditions at 37°C. Three concentrations of ZnO nanoparticles (0.005, 0.05, and 0.5 mg/ml) were tested, and a significant inhibitory effect was observed at a concentration of ZnO nanoparticles of 0.5 mg/ml (but not at 0.005 or 0.05 mg/ml), as shown in Figures 4, 5. After treatment with 0.5 mg/ml ZnO nanoparticles, the examination of the mixed cultures (*C. jejuni* with either *P. aeruginosa* or *E. coli*) by SEM indicated that the morphology of the bacteria was no longer the same as the morphology of the mixed cultures without ZnO, as shown in Figure 6.

## Discussion

The most intensive formation of biofilm by *C. jejuni* was observed in this study under aerobic conditions. Additionally, after 48 h under aerobic conditions, *C. jejuni* could not be cultivated, as *C. jejuni* entered a VBNC state (shrinking and losing its spiral shape) under aerobic conditions. However, when co-cultured with *E. coli* or *P. aeruginosa*, the survival rate of *C. jejuni* increased due to the presence of *P. aeruginosa* or *E. coli*. This makes the environment more favorable, by lowering the oxygen level and altering the secondary metabolite levels (Wu et al., 2016). Biofilm formation can increase the ability of bacteria to survive in adverse environments.

It is complicated to remove biofilms because of the sophisticated biofilm regulatory mechanisms involving many genes (Karki et al., 2019) that underlie biofilm formation (Nobile and Mitchell, 2005; Sakuragi and Kolter, 2007; Arenas and Tommassen, 2017; Shukla et al., 2017). Previous research showed that only nanosized ZnO particles had an antibiofilm effect (Alves et al., 2017), mainly because ZnO nanoparticles are small and have a large specific surface area and high oxidation ability, and their effects against biofilms are attributable to the generation of reactive oxygen species on their surface (Zheng et al., 2014; Setyawati et al., 2015).

When the ZnO concentration was 0.005 and 0.05 mg/ml, there was no biofilm inhibition effect, and the biofilm even slightly increased. The inhibition effect only appeared when the ZnO concentration was 0.5 mg/ml. It was presumed that the biofilm offered protection against the effects of the lower ZnO concentrations (Ouyang et al., 2017). Biofilms can slightly increase (up to a certain point) when harmful substances stimulate the protection mechanisms of the bacteria, but this does not work if the stress is beyond the regulation ability of the biofilm, and so the high-concentration ZnO nanoparticles had an obvious inhibition effect (Ouyang et al., 2018). With the development of high-throughput sequencing, it will be easier to identify associations between *C. jejuni* genotypes and biofilm phenotypes in the future, and this will provide new ideas to control persistent biofilms.

## Conclusion

This is the first study of microaerobic bacteria (*C. jejuni*) co-cultured with aerobic bacteria (*P. aeruginosa*) and facultative anaerobic bacteria (*E. coli*). Based on SEM, *C. jejuni* retained its normal spiral shape under aerobic conditions for 48 h by forming crosslinks with the aerobic and facultative anaerobic bacteria, which might explain how the microaerobic bacteria *C. jejuni* can survive outside hosts and how it has become a leading cause of foodborne diarrheal illness. Additionally, 0.5 mg/ml ZnO nanoparticles clearly inhibited biofilm formation, which represents an exciting new approach for developing antibacterial products in the future.

## Data Availability Statement

All datasets generated for this study are included in the article/supplementary material.

## Author Contributions

XZ, QW, MC, LX, XW, and JZ contributed to the conception and design of the study. XZ, XN, and ZM organized the database. XZ, SW, and QW performed the statistical analysis. XZ wrote the manuscript. JZ, QW, JW, YD, and YZ supervised the manuscript. All authors contributed to the manuscript revision and read and approved the submitted version of the manuscript.

## Conflict of Interest

XZ is employed by Hong Zheng Dao (China) Traditional Chinese Medicine Research Company Ltd. The remaining authors declare that the research was conducted in the absence of any commercial or financial relationships that could be construed as a potential conflict of interest.
